# Developing a Regional Strategy for Older Adults Living With Frailty: Recommendations From Patients, Family Caregivers and Health Care Providers

**DOI:** 10.5334/ijic.6438

**Published:** 2022-09-02

**Authors:** Jacobi Elliott, Melissa Koch, Miranda McDermott, Veronica Sacco, Paul Stolee

**Affiliations:** 1 School of Public Health Sciences, University of Waterloo, 200 University Ave W, Waterloo, ON, N2L 3G1, Canada; 2 Lawson Health Research Institute, 750 Base Line Rd E, London, ON, N6C 2R5, Canada

**Keywords:** frailty, care integration, aging, health care, regional strategy, community consultation

## Abstract

**Introduction:**

Health care organizations are increasingly recognizing the need to integrate the health care system to better care for older adults. We partnered with a local health centre to inform the development of a *Regional Frail Senior Strategy* for Southwestern Ontario, Canada.

**Methodology:**

Interviews were conducted with 12 older adults (65+, with chronic conditions) and family caregivers. 44 interviews were also completed with health care providers from across the region. To engage with a range of stakeholders on the strategy, four feedback fairs were hosted. Interviewees emphasized the importance of person and family-centred care, integration of health care services, issues of access, and further training and education for health care professionals. Findings and stakeholder feedback were synthesized into 14 recommendations.

**Discussion:**

The data and recommendations outlined in this paper informed the development of the frailty strategy for a region in Ontario. Participatory methods and stakeholder engagement identified pertinent themes related to enhancing care for older adults with frailty.

**Conclusion:**

The creation of a frailty strategy is imperative in recognizing and responding to the needs of older adults with complex conditions. Our approach may be relevant to other organizations and health systems interested in developing their own regional frailty strategies.

## Introduction

Health care for older adults living with frailty is challenged by poor system integration and coordination, as well as a shortage of specialized geriatric services [1,2]. One study using the Frailty Index and a sample from the Canadian Community Health Survey, approximated that the prevalence of frailty in community-dwelling adults aged 65 and older in Canada is 24% [3]. These levels of frailty are problematic within an aging population and health care system that currently works to reactively treat individual acute health issues [4].

Defining frailty remains elusive [4,5,6,7,8,9,10,11,12,13,14,15,16,17,18,19] and while a specific definition of frailty is not provided in this study, our understanding of frailty will be informed by common elements found in a number of frailty definitions which usually include words such as decline and vulnerability [5,10,11,12]. Numerous definitions also mention reduced or loss of physiological reserve across multiple systems, including physical, mental, social, emotional and biomedical domains [7,9,10]. Often this can lead to the inability or increased difficulty in managing stressors (e.g., acute, every day, environmental), leaving the individual living with frailty vulnerable to adverse health outcomes [8,9,11,13].

As the population ages, health care providers and organizations are increasingly recognizing the need to intentionally and systematically address frailty [17]. An increasing prevalence of chronic conditions requires a system more focused on holistic health and wellness, prevention, and integrated care models that support improved patient outcomes [4,18]. This has led to the rise of a number of Canadian and international frailty strategies as well as other programs designed to support older adults living with frailty and their caregivers. Examples include: the SIPA model in Quebec [19], COPA in Paris, France [19], the GAIN model in Central East Ontario [20], the ACE strategy in Toronto, Ontario [21], the Guided Care Model in Washington, D.C. [22], and the CARES model in Nova Scotia and British Columbia [23].

There is much to be learned from these existing strategies and programs, but each health care organization must ensure their strategies are reflective of their specific context and the particular needs of their respective patient populations [24]. Our research group partnered with St. Joseph’s Health Care London to inform the development of a *Regional Frail Senior Strategy in the South West Region of Ontario*, the region herein referred to as the ‘South West’. In order to develop the strategy, our research question focused on understanding the current Specialized Geriatric Service pathways across the South West. This paper outlines a case study that lays the groundwork for developing integrated care for older adults living with frailty in the region. The strategy was informed using principles of co-design by capturing the experiences of health care providers, patients and caregivers, and then engaging stakeholders to identify priorities and make recommendations.

## Methods

To develop the regional strategy, we first conducted an extensive literature review on existing frailty strategies, frameworks and models (reported elsewhere; [25]). The reviewed literature indicated that a prominent goal of supporting individuals with complex needs is providing a more holistic care approach [25]. Informed by this review, we conducted in-depth interviews with a range of stakeholders who had lived experience of the health care system, as well as held a series of community consultations. Our work, which was rooted in the perspectives of older adults with frailty and their caregivers, was guided by literature on patient/caregiver engagement [26] and person-oriented research [27]. This present analysis will focus on the interviews, community consultations, and resultant recommendations. Ethics clearance was obtained through the University of Waterloo Office of Research Ethics (ORE # 31598).

### Context & Setting

This research was conducted in the South West region of Ontario, Canada which consists of five ‘sub-regions’: Elgin, Oxford, Grey Bruce, Huron Perth, and London Middlesex [28]. We endeavoured to collect data and represent all regions in this work. As of 2021, it has been estimated that over 20.5 per cent of the population in the South West is over the age of 65, which is approximately 200,000 people [29]. In Canada, 25% of individuals in this group (65+), and 50% of individuals over 85 are frail [29]; this regional data highlights the immediate need for a more integrated and coordinated approach to provide seamless care for older adults. The South West region includes rural and urban areas, academic centres and health care settings that serve diverse communities.

This work was overseen by a Project Advisory Group at St. Joseph’s Health Care London which included the Director of Specialized Geriatric Services, Director of Specialty Mental Health Programs, a Geriatrician, a Geriatric Psychiatrist, Coordinator of the Regional Frail Senior Strategy and a Quality Improvement/Knowledge Translation Facilitator. This committee provided our group with relevant regional reports and papers, identified potential participants, and shared their knowledge of the broader health care system in the South West. They also reviewed our group’s work and provided ongoing feedback.

### In-depth Interviews

Semi-structured interviews were conducted with health care providers, older adults, and caregivers across the South West region to understand strengths of the health care system and to identify areas for improvement [please see Appendix 1]. Interviews lasted approximately thirty to sixty minutes and purposeful strategies for sampling were used [30]. Project and committee members identified health care stakeholders who would be able to speak to processes for referring to and conducting comprehensive assessments, services, and supports for frail older adults and caregivers, and who had knowledge of the broader health care system in the South West LHIN (LHINs were regional health authorities in Ontario).

### Recruitment

Providers were recruited by the committee, principally via email, and were eligible to participate in the study if they were providing care to older adults in the South West region or held a leadership position for an organization responsible for providing care to older adults. Non-English speaking providers were excluded for the purposes of this study. Forty-four interviews were conducted with health care providers from across the region. Their roles included physicians (n = 10), nurses (n = 14), health care administrators (n = 15), and other health professionals (n = 5). Twelve interviews were completed with older adults and caregivers across the region. Eligible study participants must have been receiving geriatric care in the South West region and included individuals with cognitive impairment. Non-English speaking older adults and caregivers were excluded. Recruited patients and their caregivers had experience managing a range of health-related issues (e.g., falls, cataracts, pneumonia, dementia, strokes, surgery, infections and anxiety). Some of the participants were able to provide dual perspectives, as they had been both a patient themselves and a caregiver to others. These individuals were identified through the Geriatric Resource Nurses, and other providers we interviewed. Providers were given a recruitment script to help explain the project to patients that are over the age of 65 and living with multiple health conditions (or a caregiver of someone who meets this description). If the patient and/or caregiver was interested and gave their permission, the providers forwarded their contact information to our research team.

### Data Collection & Analysis

Two research associates conducted interviews with health care providers and older adults by telephone and in-person. Upon reviewing the study’s letter of information and obtaining consent, providers and older adults/caregivers were asked to comment on the strengths and gaps related to care for frail older adults in the region.

Interviews were audio recorded and transcribed verbatim by trained research assistants. Data were anonymized and entered into NVivo 11 to support qualitative analysis [31]. The two researchers who conducted the interviews and two research assistants analyzed the data line-by-line. A reflexive thematic analysis was completed to generate themes [32]. Our approach was guided by the following process proposed by Braun & Clarke [33]: familiarizing yourself with the data, generating initial codes, generating themes, reviewing themes, and defining and naming themes. Reflexive practices [30] were incorporated throughout the stages of data collection and analysis, such as memoing and debrief meetings between members of the research team to develop a richer reading of collected data [32].

### Community Consultations

As a final step to inform the regional frail senior strategy in the South West, community consultation events were held in four of the five sub-LHIN regions (Grey Bruce; Huron-Perth; Oxford; Elgin) where the findings from the literature review and interviews were showcased. In total, across the four events, 94 individuals came out to share their perspectives and provide feedback on our work. These individuals included health care providers, patients and caregivers of all ages, and members of the public. The four events ranged from 3.5 to 5 hours and were held in a large community space. Attendees were able to walk around to different stations that showcased information/results on presentation boards. Attendees could leave their thoughts on sticky notes and each station had different guiding prompts for participants to think about such as: *What are your thoughts on what we found?, How can we solve some of the current health system challenges?, What successful or positive experiences have you had in the health care system?, What models/programs/services/initiatives can we learn from?* Researchers and the regional frail senior strategy team members spoke with attendees throughout the events and recorded field notes about these interactions. In addition to the field notes, comments left on sticky notes were all transcribed and analyzed. The data collected as part of the community consultations supported developed themes and provided additional description prior to finalizing themes.

## Results

### Interview Themes

Across all 56 interviews, twelve with older adults and caregivers and forty-four with providers, several common elements among themes emerged. Both groups of participants emphasized the critical importance of care that takes into consideration the needs and goals of older patients and their families, issues of access, and the importance of further training and education for health care professionals. Key themes are presented in Tables 1 and 2. Please refer to Appendix 2 for example participant quotes.

**Table 1:** Health care provider interviews-main themes.


THEME	DESCRIPTION

Importance of Providing Care Grounded in Person and Family Centred Approaches	Health care providers mentioned that an important component of providing care for older adults is being patient and family-centred. This includes incorporating the perspectives and goals of those who are receiving care into health care discussions, as well as offering flexibility in service provision to support the diverse needs of patients and their caregivers.

Cross-Sectoral Communication and Coordination	While health care providers commented that collaboration is improving, gaps in communication and information sharing persisted especially between providers caring for frail older adults across different sectors/levels of care. Lack of coordinated service provision was also raised as an issue.

Improving Navigation Through Increased Understanding of the System	An idea that consistently arose throughout interviews with various healthcare providers was the need for improved understanding of available resources throughout the system, which would in turn allow them to provide patients and caregivers with more information regarding available services and avoid duplication of efforts.

Accessibility of Care	Participants identified frustrations surrounding wait times for services (i.e., specialized services, general geriatric appointments, community supports, and long-term care beds), which created a barrier to accessing care. In an effort to make health care more accessible, primary health care providers spoke about the importance of visiting patients in their homes and felt that ongoing efforts must be made to strengthen home supports. Issues around geography also impacted accessibility of care; inequity in availability of resources existed across different regions in the South West, such as inadequate transportation options.

Challenges with Capacity	Challenges associated with human resources was discussed by participants, which encompassed a shortage of geriatricians, health care providers with specialized geriatric training, family physicians and personal support workers.


**Table 2:** Patient and caregiver interviews-main themes.


THEME	DESCRIPTION

Importance of Providers Who Take into Consideration Unique Needs of Older Adults	Patients and caregivers described their health care experiences more positively when they felt that health care providers demonstrated compassion and listened to their needs, especially for older adults with cognitive impairment.

Need for Support Services and Information for Caregivers	Caregivers broadly felt that they needed additional support and education to better meet the needs of their loved ones, especially for young people in caregiver roles. Beneficial supports and services included: respite services, day programs and caregiver support groups. However, accessing information could be difficult and contributed to increased caregiver burden.

Patient Advocacy While Navigating the System	Participants talked about the importance of having a family member or friend who was familiar with the system and could be an advocate. At times, both caregivers and patients did not feel empowered to voice their concerns, with some caregivers finding it difficult to advocate for themselves and set boundaries in their roles.

Providers’ Knowledge of Community Supports and Training in Geriatrics	While some participants spoke positively about geriatric specialists in the community and primary memory care clinics, others felt that providers did not have sufficient knowledge of available geriatrics community services and adequate training in responsive behaviours.

Addressing Gaps in Home Care Services	Many patients and caregivers had positive experiences healthcare providers and services that were offered in the home, but a shared sentiment was there was room for improvement, especially at a policy level. High rates of staff turnover, staff changes when care transitions occur and limited services that are offered in the home were mentioned as gaps.

Allocation of Funding and Resources to Better Support Older People	Participants talked about the allocation of funding and resources in our health care system to create sustained improvements in the care of older adults. Some suggestions included the allocation of case managers in primary care and providing more resources for mental health and addiction.


### Community Consultations

**Figure 1:**
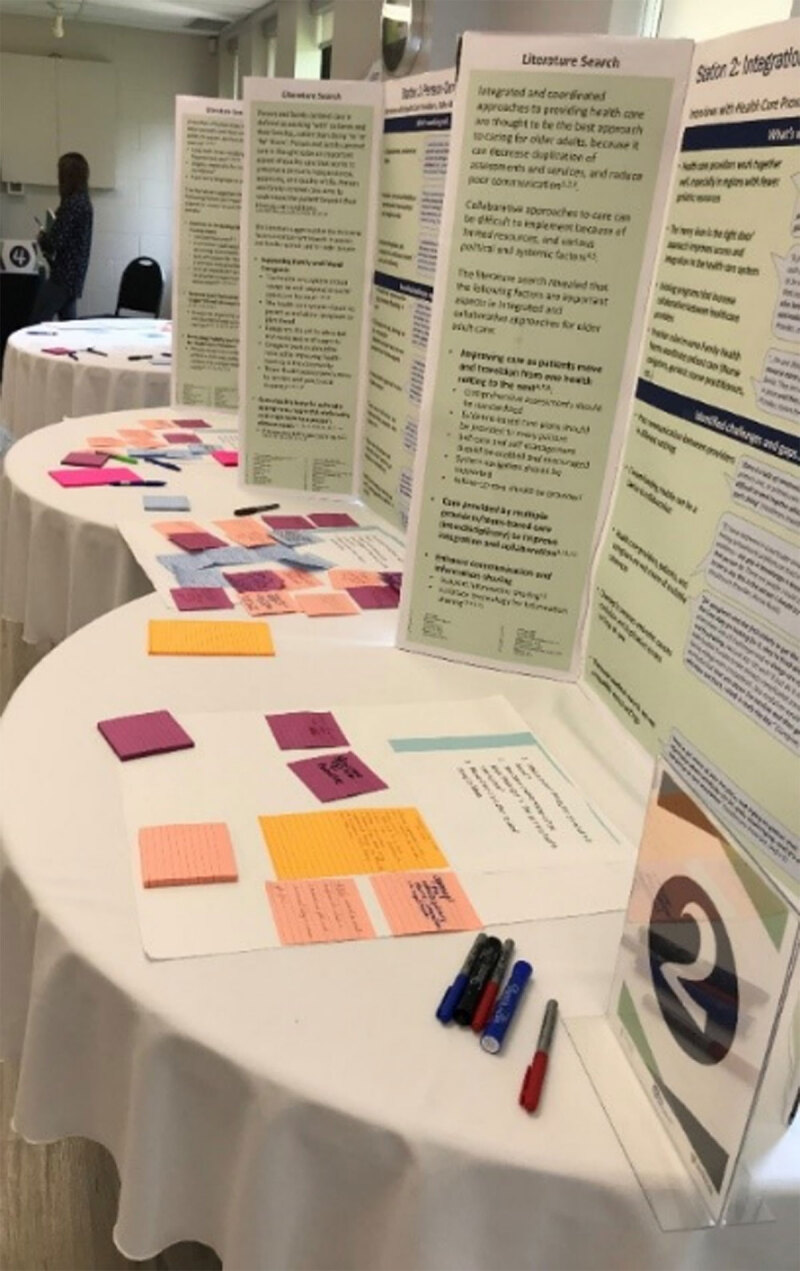
Community consultation feedback fair stations.

The community consultations, which we titled “feedback fairs”, were successful in obtaining input from a variety of health providers, older adults, caregivers and members of the public (see Figure 1). At all four consultations, participants agreed that improvements need to be made with communication, navigation and education in the health care system. Many participants also talked about the importance of improving transportation services and health services in rural settings.

This feedback functioned as a form of ‘member checking’ by ensuring that our analysis and understanding of the interview data was reflective of stakeholder experiences [34]. This ensured methodological rigour alongside other employed strategies, such as memo writing during data collection and analysis. Secondly, it allowed us to synthesize our results and begin to identify recommendations.

### Recommendations

Building off the interview data, literature review and community consultations, we synthesized our findings into several key recommendations for the frailty strategy (summarized in Table 3).

**Table 3:** Recommendations for the regional frailty strategy.


RECOMMENDATION	SPECIFIC ACTIONS

**Promote care that is flexible based on the patient’s individual needs**	Patients’ cognitive ability, needs and preferences should be considered when providing care; especially during care transitions

**Improve education for caregivers and patients**	Provide one clear resource for caregivers and patients to access information about programs and services; ensure this highlights which services accept self-referrals

**Increase access to primary and specialized care for older adults across the region**	Expand the opportunities for geriatric specialists to work within and across the region; this may require a plan to attract and retain Consider how existing nurses and nurse practitioners can improve timely access to care

**Continue to leverage telemedicine and telehomecare technologies**	Use of telehomecare evaluated at the local level (rural/underserviced communities) and based on individual need (e.g. mobility needs), as well as during transitions home from hospital

**Develop localized geriatric teams in each sub-region**	Each team should have a system to collaborate with primary care, home and community care, mental health support services and other community support services Expand the role of geriatric resource nurses

**Build expertise and improve quality of geriatric care across healthcare providers**	Develop a regional frail senior education plan; leverage existing educational materials and advocate for upstream changes to improve training in geriatrics

**Improve transportation services in rural regions**	Ensure older adults are able to access non-profit transportation that meets their accessibility needs

**Improve integration of services across the care continuum**	Develop a coordinated intake for all services for frail older adults within each sub-region

**Improve alignment and integration of specialist care**	Work towards a model of improved alignment and integration of resources (including geriatric psychiatry, geriatric medicine, and other specialists) to provide comprehensive specialist assessments

**Develop innovative funding models for programs and services**	Consult with healthcare providers to understand how funding could effectively incentivize collaboration and shared care

**Improve health care information sharing technologies in the region**	Consider a single patient chart that is accessible to all health care providers and community support services

**Develop and use consistent language to describe services and roles**	In consultation with stakeholders, including patients and caregivers, consistent language should be agreed upon and used throughout the region

**Develop one clear information source for providers to access information about resources**	Provide one number for patients/caregivers and providers to call, for each region, to receive assistance navigating and information about supports and services. Leverage existing websites to highlight this information

**Expand support for patients and caregivers who are navigating through the system**	Consider the use of system navigators and/or peer-support services


## Discussion

Collectively, the data and recommendations summarized here formed the frailty strategy for the South West region in Ontario, Canada. These recommendations have served as the foundation for the region, to guide initiatives and organizational changes aimed at improving care for frail older adults. With an emphasis on participatory methods and stakeholder engagement, this strategy is emphatically and intentionally grounded in the lived experiences of older patients, their caregivers and health care providers [35,36]. In the creation of this strategy, our research has created a direct link between the voices of key stakeholders, including older adults themselves, and the creation of policies and practices to improve care.

Across all phases of this study, person-centredness, access to care, resources and capacity, system navigation, coordination of care, strengthening home-based supports and communication and collaboration arose as universally important themes for improving the care of older adults living with frailty. These themes align with both local and international guidelines and frameworks on providing integrated care for older adults with complex conditions [37,38,39]. Similar to our overarching themes, Horgan and colleagues [37] emphasize integrated care models rooted in collaboration, cross-sectoral partnerships, older person-centred care, improved communication and coordination, and integrated community and home-based services. Developed themes from this project are echoed in the WHO’s implementation framework, including coordinating services delivered by multidisciplinary providers and orienting services towards community-based care [38]. Our recommendations speak to these themes, with a focus on actionable next steps. Given the current funding context, it was prudent to focus on recommendations and actions that built on or leveraged existing roles and programs. Unlike established and validated programs to support older patients and caregivers in the United States [40,41], it would not have been feasible to propose, for example, adding personnel.

With respect to existing personnel, both health care providers and patients and their caregivers recognized the need for improved training in gerontology. Providers are seeking enhanced training in geriatrics, a topic that is poorly represented in most health care education programs across the country [42]. Recent estimates indicate that there are fewer than three hundred specialist physicians in geriatrics in Canada, which is insufficient [43]. Meanwhile, approximately three-quarters of all health care hours in Canada is spent with older adults requiring complex care [44]. Within this context, we need to focus on enhanced geriatric training for the host of other individuals providing care, including but not limited to nurses, personal support workers, allied health professionals and health care administrators.

The principles of the Canada Health Act provide for: public administration, comprehensiveness, universality, accessibility and portability [45]. Alarmingly, our data, in particular data from older patients and their caregivers, highlighted significant issues of accessibility. Frail older adults with disabilities, living in rural regions and without their own financial means to reach care providers, are not necessarily accessing the care that they need. While our recommendation to improve transportation may appear to be outside the purview of health care, comprehensive frailty strategies must place a particular emphasis on health equity and ensuring access for those who are most vulnerable.

Findings from this work have provided evidence for adopting regionally integrated and coordinated approaches for older adults with complex conditions. A regional strategy for older adults at-risk of, or living with, frailty, could assist in guiding local policy and practice decisions surrounding funding allocation and building effective partnerships to improve the care experience of older adults and their caregivers. The information gathered and proposed recommendations have been synthesized and subsequently translated into the South West Frail Senior Strategy’s *Strategic Priorities 2019-2022*. Next steps include implementing and evaluating new processes for coordinating specialized geriatric care, including mental health services.

One limitation arising from this case study is the highlighted findings and recommendations are derived from the priorities and needs of the South West LHIN. While this may influence the generalizability of results to other contexts, organizations interested in developing a regional frailty strategy may learn from the processes and approaches used. Secondly, this work only highlights perspectives from the health care system. Traditionally, health system improvements have not engaged other sectors, such as social care; this should be considered in future work. Furthermore, we knew that it was crucial to focus data collection on older individuals and their caregivers with lived experience of frailty [36]. This population, however, was difficult to access and ideally, we would have recruited a larger sample of older adults living with frailty.

## Lessons learned

- A regional approach rooted in understanding the local context and programming is imperative when creating strategies and policies for older adults with frailty.
- Recommendations for a regional frailty strategy are most feasible and effective when focused on re-organizing and re-distributing current resources, rather than adding additional resources.
- Engaging older adults and caregivers in the development of regional frailty strategies ensures that the voices and perspectives of individuals impacted most by the strategy are included.
- To support equitable participation in this work, multiple approaches were used to collect the experiences and perspectives of different stakeholders and community members.
- The community consultations are an innovative approach that allowed for further engagement with members of the community and may be of interest to other researchers as a methodological approach.

## Conclusion

This work has supported the development and ongoing implementation of the *Regional Frail Senior Strategy* in the South West region of Ontario, and has the potential to inform frailty strategies for other health organizations in Canada and beyond. Health care organizations have been slow to respond to the realities of an aging population [46] and the creation of a frailty strategy grounded in principles of integrated care (i.e., person-centred approaches, cross-sector communication and collaboration, improved system navigation and access to care) is an important step in formally recognizing and then responding to the needs of older patients with complex care needs. Other agencies seeking to develop a strategy for older patients with frailty can build from both the recommendations and processes outlined in this manuscript.

## Additional Files

The additional files for this article can be found as follows:

10.5334/ijic.6438.s1 Appendix 1. Interview/focus group guides.

10.5334/ijic.6438.s2 Appendix 2. Themes with Participant Interview Quotes.
