# Supplementary material for: Developing a Regional Strategy for Older Adults Living With Frailty: Recommendations From Patients, Family Caregivers and Health Care Providers
Source: Int J Integr Care. 2022 Sep 2;22(3):13. doi: 10.5334/ijic.6438 (PMC9438459; doi:10.5334/ijic.6438)
Supplement: Appendix 1. — Interview/focus group guides. [file ijic-22-3-6438-s1.pdf]

Appendix 1- Interview/focus group guides

i. **Patients/Caregivers**

CONTEXT QUESTION

1. Can you please tell me about your [or your family members'] current health care experiences
    a. *Supports you are receiving*
    b. *Health care providers you are seeing or waiting to see?*
    c. *Health conditions you are experiencing*

    OR
2. Can you please tell me about the services, support and care that you are currently receiving? (e.g. *Probe: specialist care, family physician, memory clinic, home and community care, other community supports)*

MAIN QUESTIONS

3. Can you tell me what brought you to {SGS}? What medical or function issues resulted in the referral
    a. Do you recall who referred you?
    b. How did you feel about the referral?
    c. How long did it take to get an appointment? *How did that make you feel?*

4. Can you please walk me through your first appointment with {SGS provider}? What kinds of questions did they ask you?
    a. How did you feel about the conversation?
    b. Can you recall what kinds of health care providers were involved? (e.g. SW, nurse, OT, PT, etc.)

5. Did the care provider give you any recommendations or suggest other referrals?

6. Thinking back on your health care journey – can you tell me about some experiences that have gone particularly well?
    a. What made this a positive experience?

7. Thinking back on your health care journey – can you tell me about some experiences that did not go so well?
    a. What made this a negative experience?
    b. What could have been done to improve this experience?
        - *Probe: if you had a magic wand and could make the SGS system better, what would it look like? Who would be involved? What resources would you need?*

ENDING QUESTIONS

8. Is there anything else you think I should know? Anything else you would like to share?

9. Is there anything else you would like to ask me?

ii. Health care providers

CONTEXT QUESTIONS

1. I would like you to share your name, the organization you work for, and your role in caring for frail seniors and their caregivers

- Can you tell me about your role (and the role of your organization/program) in the care for frail seniors?
- Can you please tell me about the services, support and/or care that you provide to frail seniors?

FOR PRIMARY CARE/COMMUNITY

2. Can you please describe to me, the specialized services available for frail seniors in your region?
   a. *Probe: SGS, home care, etc.*

3. Please walk me through your current process for **assessing** and **referring** an older adult who needs a more comprehensive assessment.
   a. *Probe: wait time? How long does it take for your patients to be seen by SGS?*

FOR SPECIALIST/SGS PROVIDERS

4. Can you please describe to me, the specialized services available for frail seniors in your region?
   a. *Probe: SGS, home care, etc.*

5. Can you please describe to me, the process of receiving referrals?
   a. Who sends referrals? *Probe: to understand different pathways*
   b. What are the next steps? *Probe: process for booking, what are wait times, etc.*

6. Please walk me through your current process for assessing a frail older adult.
   a. What happens next? *Probe: what does CGA look like? How long does this process take?*
      i. *Probe: connect back to primary care?*

MAIN QUESTIONS

7. What do you feel is currently being done particularly well in the way that care/support is provided to frail seniors and their caregivers as they move throughout the health care system?

8. What are the gaps in the current [local] system in caring for frail seniors?

- Are there any gaps in the current system? If yes, what are they?
- *Probe: Lack of SGS services? Need more interdisciplinary team members? Need more resources? Etc.*

9. What do you think could be done to improve the care of frail seniors and their caregivers as they move throughout the health care system from primary care to SGS for comprehensive geriatric assessments?

- Is there anything about the way that support/care is provided to frail seniors and their caregivers as they move through the health care system that could be improved?
- *Probe: if you had a magic wand and could make the SGS system better, what would it look like? Who would be involved? What resources would you need?*

ENDING QUESTIONS

10. Is there anything else you think I should know? Anything else you would like to share?

11. Is there anything else you would like to ask me?
