# Supplementary material for: Developing a Regional Strategy for Older Adults Living With Frailty: Recommendations From Patients, Family Caregivers and Health Care Providers
Source: Int J Integr Care. 2022 Sep 2;22(3):13. doi: 10.5334/ijic.6438 (PMC9438459; doi:10.5334/ijic.6438)
Supplement: Appendix 2. — Themes with Participant Interview Quotes. [file ijic-22-3-6438-s2.pdf]

Appendix 2- Themes with Participant Interview Quotes

Main Themes with Example Quotes: Health Care Providers

Theme	Example Quotes
**Importance of Providing Care Grounded in Person and Family-Centred Care Approaches**	*"…**communication, coordination and making sure that it's done in a way that really is client and family centred…we have a family representative who sits on our committee**, who reminds us about the importance of patient and client and family-centred care from the perspective of being on the receiving end."* (Healthcare Director, Huron Perth)
**Cross-Sectoral Communication and Coordination**<br>•	*"**There is a lack of communication** between the community and primary care, or primary care and the hospital…**So it's really difficult to work together when you don't know what the other guy's doing.**"* (Healthcare Provider, London Middlesex)<br><br>"**The other gap I think is just in coordination of services**. Because there are so many great things that are happening in London, and depending on who your provider is, you may or may not get linked into some of these great services." (LHIN representative)
**Improving Navigation Through Increased Understanding of the System**	*"**People still don't know what the system is,** and they don't know where to direct their patients…their patient's been sent to the memory clinic, has been sent to our team and has been sent to BSO [Behavioural Supports Ontario] all at once because primary care didn't know where to send them…**we find that we end up with multiple players involved and people telling their stories over and over again.**"*(Healthcare Provider, Grey Bruce)
**Accessibility of Care**	*"**We have gaps in access.** So **access to primary care** for seniors, whether it's **transportation to a physician's office**, whether it's **the physician or nurse practitioner providing house calls**, frail seniors, **just getting an appointment when they need the appointment**. What everybody else faces, **magnify that if you're hearing impaired or you're visually impaired, or mobility is an issue, the access is magnified in terms of complexity and gaps.**"*

	(Healthcare Director, SW LHIN)
**Challenges with Resource Capacity to Serve Complex Older Adults**	*"When we look at, previous DELPHI consensus, we think that you need one geriatrician to every ten thousand older adults**…And so right now they're like woefully understaffed in that region, obviously because there's so few geriatricians in Canada.**"* (Healthcare Provider, Huron Perth)

Main Themes with Example Quotes: Patient and Caregivers

Theme	Example Quotes
**Understanding older adults' needs and preferences**	***"I feel that doctors need to be more in tune****… I have one friend who's looking after somebody and when she goes to the doctor, she gets exactly ten minutes. And I said, 'oh you've got to be joking'…"* (Patient & Caregiver, Huron Perth)
**Need for Support Services and Information for Caregivers**	*"I went on my own and contacted the Alzheimer's Society of London Middlesex. **And that was where the doors opened and I got a lot of help…Tremendous amount of navigating the health care system, because it's not easy.**"* (Caregiver, London Middlesex)
**Patient Advocacy While Navigating the System**	*"My experience wasn't bad…**but I'm in the system, I know how to advocate**, I notice this stuff. I say this every time I get an opportunity… **how [do] people who don't know the system navigate this nightmare**…"* (Caregiver, Oxford)<br><br>*"…**if you don't have a family member or close friend to advocate for you… I think you have a serious problem in the system**, I really do. Like, if you're in the hospital….if you were there and you had no family or friends nearby, I think it'd be very, very different experience, because it really helps. **Especially if you have cognitive issues, that's what my father has. You really need to have someone who's going to stand up for you** and say, "Well, why can't we do this or that?" Or, you know, "Are we looking into this? Or… because I think you'll sort of get lost in the system…. I think that you really, really*

	have to have someone advocate for you…" (Caregiver, Grey Bruce)
**Providers' Knowledge of Community Supports and Training in Geriatrics**	*"This is a problem that's going to only get worse… and* ***family docs are the gatekeepers of the health care system. They need to be more informed about various supports in the community****…".* (Caregiver, London Middlesex)
**Addressing Gaps in Home Care Services**	*"…you know,* ***I'm just saying overall, the home care system is good in Ontario, but there's really…there's a lot of room for improvement.*** *We have a great case manager that we have who… has been amazing, and that makes a real difference. Having someone who I know is there as a good advocate for what we need and… she has her constraints obviously.* ***There's only so much time allotted to any individual patient in the home care system within a month.*** *So you know, there's definitely the limit as to what's possible for… that patient and the patient's family or patients caregivers…to get from the system. They need to do a… I don't mean the CCAC or LHIN, but the overall Ministry of Health in Ontario, they have a long way to go when it comes to taking care of seniors in Ontario."* (Caregiver, Grey Bruce)<br><br>*"****One of the challenges with CCAC…was the staff turnover.*** *I think it's a pretty stressful environment in which to work. So I think there was a lot of social workers who you'd speak to and then they'd be off on leave, they would have left and then you get a new social worker* ***so the person has to start all over again****."* (Caregiver, London Middlesex)
**Allocation of Funding and Resources to Better Support Older People**	*"…they need to put a lot more money into the system than they do, but that's easier said than done…and they need to spend it more wisely than they do…just because we have more people aging…****they're just throwing band-aid solutions…there's really not a lot of…where do we see the system going and can we really make a change for the positive?"*** (Caregiver, Grey Bruce)<br><br>*"****I feel that the government could save a lot of***

	money if these family health clinics had their own case managers*…because they know the clients, they know the family, they know everything. It's not like a stranger being interviewed to come in who knows nothing about you.* ***And I think that they would get better care, I think there would be less emergency care****… the clinic could have their own case managers, they would have care conferences and I think things would get picked up a lot easier and I think families would be far more conducive to when the time comes that they have to go on to something else."* (Patient/Caregiver, Huron Perth)
